# Advances in high-throughput methods for the identification of virus receptors

**DOI:** 10.1007/s00430-019-00653-2

**Published:** 2019-12-21

**Authors:** Sarah V. Barrass, Sarah J. Butcher

**Affiliations:** Faculty of Biological and Environmental Sciences, Molecular and Integrative Bioscience Research Programme and Helsinki Institute of Life Sciences, Institute of Biotechnology, University of Helsinki, P.O. Box 56, 00014 Helsinki, Finland

**Keywords:** Receptor identification, Mass spectrometry, Genome perturbation, Virus

## Abstract

Viruses have evolved many mechanisms to invade host cells and establish successful infections. The interaction between viral attachment proteins and host cell receptors is the first and decisive step in establishing such infections, initiating virus entry into the host cells. Therefore, the identification of host receptors is fundamental in understanding pathogenesis and tissue tropism. Furthermore, receptor identification can inform the development of antivirals, vaccines, and diagnostic technologies, which have a substantial impact on human health. Nevertheless, due to the complex nature of virus entry, the redundancy in receptor usage, and the limitations in current identification methods, many host receptors remain elusive. Recent advances in targeted gene perturbation, high-throughput screening, and mass spectrometry have facilitated the discovery of virus receptors in recent years. In this review, we compare the current methods used within the field to identify virus receptors, focussing on genomic- and interactome-based approaches.

## Introduction

Viruses are intercellular pathogens dependent on their host’s cellular machinery for replication. To infect the host, the virus must first gain entry into the cell, breaching the cell’s primary barrier to infection, the cell membrane. The entry process is initiated by the interaction between viral attachment proteins and host cell surface structures. Host cell surface structures can act as either attachment factors, which localise the virus at the cell surface, or entry receptors, which actively initiate virus entry (reviewed in [1]). In the literature, receptors are referred to as molecules that can mediate attachment, entry into the endocytic compartment, entry from the endocytic membrane into the cytosol, and uncoating. Uncoating leads to the release of the viral genome from its protective capsid structure, and proceeds in a stepwise fashion under the guidance of cellular cues [2]. In this review, we use a narrower definition of a receptor, referring to those molecules that are actively involved in the entry of viruses into eukaryotic cells.

Interactions with host attachment factors are often electrostatic and non-specific, only acting to localise the viral particle before the recruitment of specific receptors required for entry. Often, these are low-affinity interactions and utilise common glycolipids and glycoproteins found on host cells, such as heparan sulphate used by tick-borne encephalitis virus [3–5]. Additionally, viruses have been shown to use different attachment factors depending on the model system used, possibly reflecting wide host and tissue tropism [3].

After the virus is concentrated onto the host cells, binding to specific entry receptors can occur. These receptors actively facilitate host cell entry by one of two processes: receptor-mediated endocytosis or receptor-mediated activation of signalling pathways, leading to virus internalisation (reviewed in [6]). Some viruses only require one receptor to facilitate entry into host cells, such as sialic acid for influenza A virus [7]. However, some viruses require co-receptors on the same cell or different receptors on different cell types to initiate infection. This is observed for measles virus, which uses the signalling lymphocyte-activation molecule and nectin-4 as receptors, and hepatitis C virus using tetraspanin, occludin, human scavenger receptor class B type I, and claudin-1 [8–13]. These receptors may be required for infection to occur, or partially redundant only assisting with viral infection. For hepatitis C, tetraspanin, occluding, and claudin-1 have been shown to be crucial for virus entry, whereas human scavenger receptor class B type I has been shown to be partially redundant [14].

Regardless of the method utilised by the virus for cell entry, virus–receptor interactions are pivotal in establishing infection. Therefore, understanding viral–receptor interactions is of significant importance from a cell biology perspective, allowing us to expand our knowledge of viral life cycles, tissue and species tropism, and pathogenesis. Furthermore, these insights can lead to the development of new antivirals, vaccines, and diagnostic technologies, combating pathogenic human viruses.

A wide range of methods have been applied over the years for the identification of viral receptors, with early studies (reviewed in [15]) using monoclonal and anti-idiotypic antibodies, solid-phase assays, and affinity purification with receptor antibodies [16–19]. However, over the last 2 decades, the field has advanced significantly due to the development of targeted gene perturbation, high-throughput screening (HTS), and the application of a new generation of high-resolution orbital mass analysers [20]. This has led to techniques such as affinity purification mass spectrometry and genetic screening, becoming more common for receptor identification. In this review, we will compare and contrast different genomic and interactome approaches utilised for the identification of host protein receptors.

## Genomic approaches

Genomic approaches have undergone significant advances over the past 2 decades, in particular through the evolution of HTS techniques. Genomic approaches can be divided into two categories: loss of function (LOF) or gain of function (GOF). LOF approaches inactivate host factors, allowing their influence on viral infections to be observed. In theory, genome-wide screening with this approach allows for the identification of all host factors, which either promote or inhibit infection. In contrast, GOF approaches introduce single new functionalities, from permissive cell lines into non-permissive ones. Virus entry can then occur if the GOF results in the expression of a functional receptor. In this review, we compare four gene perturbation techniques: complimentary DNA (cDNA), RNA interference (RNAi), random insertional mutagenesis, Clustered Regularly Interspaced Short Palindromic Repeats/CRISPR associated protein 9 (CRISPR/Cas9) knockouts, activation (CRISPRa), and interference (CRISPRi). Currently, CRISPRa and CRISPRi have not been reported to our knowledge in any receptor identification reports, although CRISPRa has been used to identify viral host factors [21]. We consider that these two approaches, which have fewer off-target effects, will most likely be very useful in the future, but as such fall out of the scope of this review [22, 23]. CRISPR/Cas9 knockouts are discussed in the “Genetic Knockouts section”.

## Genetic knockdowns

**Fig. 1:**
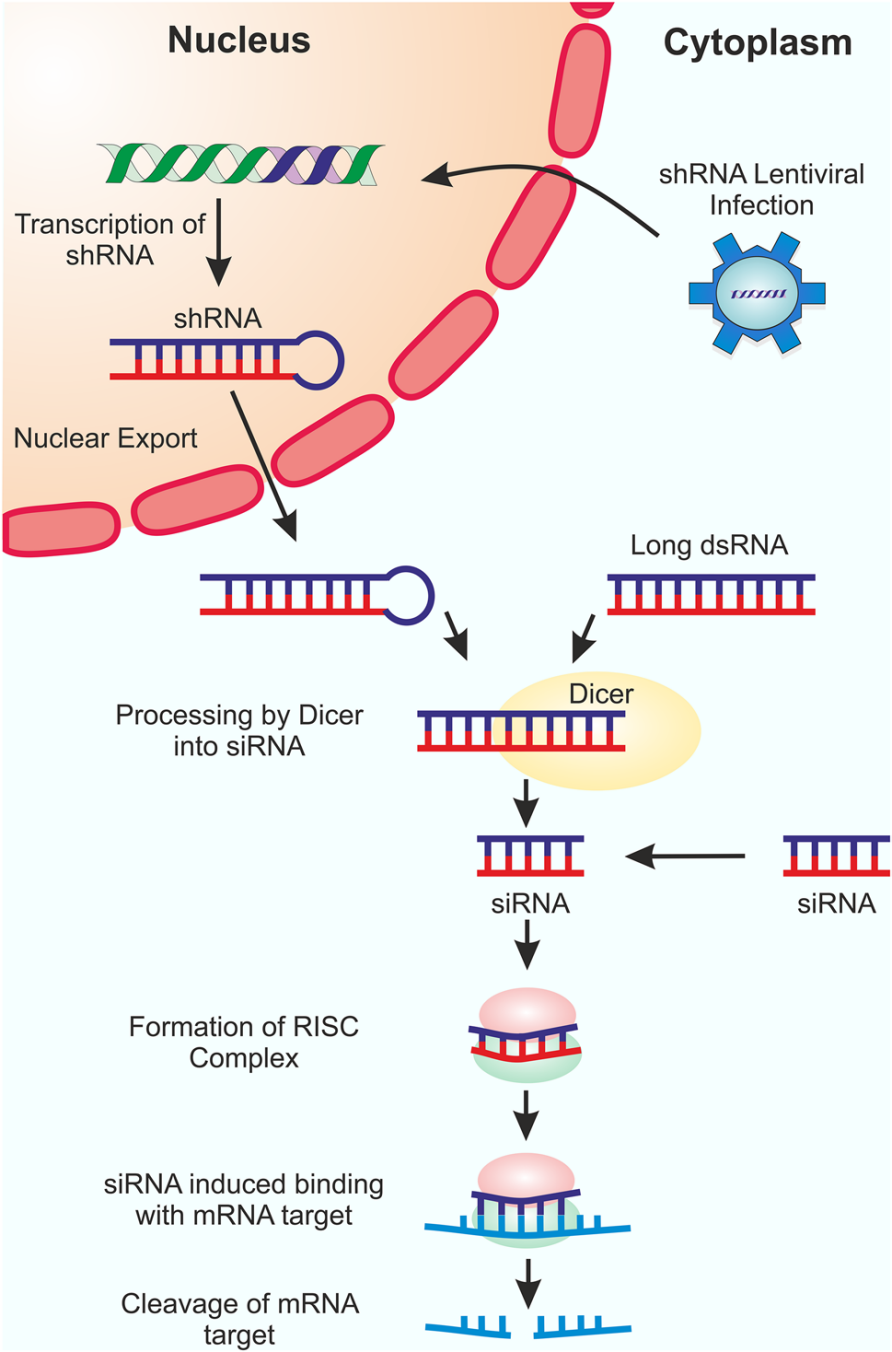
Schematic representation of RNAi silencing mechanism. For shRNA LOF screening, a lentiviral vector or plasmid encoding the shRNA (blue) is first integrated into the cellular DNA (green). The DNA is then transcribed by RNA polymerase III into shRNA and the shRNA exported into the cytoplasm by Exportin 5. For dsRNA and siRNA LOF screening, the interfering RNA is directly transfected into the cells. Both shRNAs and dsRNAs are processed in the cytoplasm by Dicer into siRNAs. The siRNAs are then loaded into the RISC and the passenger strand removed from the guide strand. The guide strand can then direct the RISC to the complementary mRNA target, where RISC cleaves the mRNA, leading to its degradation

Genetic knockdowns can be produced using RNA interference (RNAi) or CRISPR interference (CRISPRi). RNAi is a common approach utilised in LOF genetic screens and has been extensively reviewed [24–29]. RNAi acts to silence genes through the temporary removal of cellular mRNA, by RNA-induced silencing complexes (RISC) (Fig. 1) (reviewed in [30]). The RISC mechanism is universal, allowing RNAi to be used in many cell lines.

There are three tools used to achieve RNAi: small interfering RNAs (siRNAs), short hairpin RNAs (shRNAs), and long double-stranded RNAs (dsRNAs). However, the use of dsRNA is limited, as its presence induces the interferon response within vertebrate systems [31]. This causes cellular protein synthesis to close down, preventing specific loss of function phenotypes from being observed. Nevertheless, dsRNAs remain a good option for work within insect cell lines (reviewed in [32]). In 2011, a genome-wide dsRNAi screen within *Drosophila* cells identified nine transmembrane genes required for Sindbis virus infection [33]. *Drosophila* natural resistance-associated macrophage protein (dNRAMP), the homologue of mammalian NRAMPs, was the only ubiquitously expressed, plasma membrane-associated protein identified. Molecular and biochemical assays demonstrated that dNRAMP yields a fivefold improvement in virus attachment and is not required in processes downstream of entry, verifying its role as a virus receptor. Furthermore, mice deficient in NRAMP2 demonstrated a 50-fold decrease in Sindbis virus infectivity, highlighting the potential of performing dsRNAi screens in *Drosophila* cells, to identify potential mammalian virus receptors.

In contrast to dsRNAs, both shRNAs and siRNAs can be used in mammalian systems. siRNAs are directly transfected into cells and can be used in transient 3–7 day screens. Ephrin receptor A2 (EPhA2) was identified as an epithelial cell receptor for Epstein–Barr virus (EBV) using targeted siRNA screening [34]. Previously, it was reported that epidermal growth factor increases EBV infectivity [35]. Pre-treating cells with epidermal growth factor and monitoring the transcription level in a microarray screen identified six upregulated genes. The initial hits were then probed using siRNAi and only EPhA2 knockdown was shown to significantly decrease infectivity. The results were confirmed with three distinct siRNAs, thus excluding off-target effects. The results were then further validated using ectopic expression of complementary DNA, CRISPR/Cas9 knockdown, and co-immunoprecipitation assays. Finally, EPhA2 was determined to act as a receptor by monitoring the rate of virus internalisation in WT, EPhA2 knockdown, and EPhA2 knockout cell lines.

shRNAs are delivered to the nucleus in a plasmid or lentivirus, and integrated into the host’s DNA, where they are then transcribed leading to the slow accumulation of shRNAs in the cytoplasm. Unlike siRNAs, shRNA expression is stable allowing for long-term screening (> 10 days). However, expression of shRNAs may be lost if the cells lose the plasmid, or if the lentivirus integration is silenced due to negative epigenetic regulation [36, 37]. Therefore, cells need to be cultured under selection for the shRNA phenotype. shRNAi has mainly been used to validate hits identified through other methods. shRNAi was performed on *Bombyx mori* embryonic cells to knockdown the cholesterol transporter Niemann-Pick C1 (NPC1) [38]. NPC1 was proposed as a potential baculovirus receptor, due to its interaction with *Bombyx mori* promoting protein which increases baculovirus production. Two unique shRNAs were used to target the NCP1 gene and a 40% reduction in NCP1 expression was achieved, leading to a substantial reduction in baculovirus infectivity. The receptor was then further validated by performing co-immunoprecipitation experiments with NCP1 and the major baculovirus glycoprotein, gp64. NCP1 was also identified as a potential receptor for Ebola virus, by genetic knockout screening in haploid cells [39]. This was validated by shRNA knockdown of NPC1 in human peripheral blood monocyte-derived dendritic cells, which yielded them resistant to filovirus infection.

A major issue with RNAi is a lack of reproducibility between similar studies [40–42] despite efforts to minimise false positives [43, 44]. This has been investigated by directly testing the degree of overlap between two identical genomic screens. Hit lists containing 513 and 1140 hits were observed for two replicate screens, highlighting the impact of off-target effects, caused by both false-positive and false-negative results [45]. False positives occur due to the partial complementarity of siRNAs to multiple mRNAs throughout the ‘seed’ region (nucleotides 2–8 of the 21 present in the siRNA sequence). When binding occurs through the seed region, this leads to translational repression and degradation of the mRNA, via the microRNA pathway leading to off-target effects [46]. False negatives can be generated by cytotoxic knockouts leading to cell apoptosis [47], functional redundancies caused by gene duplication preventing phenotypic manipulation by single gene knockdowns [48], variation in experimental conditions [49], and systematic errors [50].

Despite these limitations, RNAi is easy to use, has a fast knockdown of 24–48 h, and uses stable reagents, making it a good choice for generating loss of function phenotypes. Furthermore, as RNAi has a knockdown efficiency which is lower than 100%, this allows the study of essential genes, as the cells can survive, whilst the virus infection may be measurably inhibited. This is a major advantage over other loss of function gene perturbation methods, such as CRISPR/Cas9 or random insertional mutagenesis genetic knockouts, where permanent null phenotypes are generated.

## Genetic knockouts

Achieving complete genetic knockdowns using RNAi is difficult. To create true genetic knockouts, which demonstrate a permanent homozygous null phenotype, deleterious gene mutations must be generated. True genetic knockouts can be generated using either random or site-specific mutagenesis.

In random mutagenesis (reviewed in [51]), cells are, for example, transduced with a retroviral genetrap vector, containing a strong adenoviral splice acceptor site and a marker gene, such as green fluorescent protein. Following transduction, genetrap vectors are integrated into introns, leading to the production of truncated mRNAs [52]. The disrupted genes can then be identified, using the integrated DNA sequences as a molecular tag. Transduction with genetrap vectors leads to biased insertion near active promoters, ensuring the generation of complete knockouts [53]. The two main drawbacks are that it generates non-universal dispersed insertions, which may prevent all loci being targeted [54], and it is inefficient in diploid cell lines, where deleterious mutations must be generated in both chromosomes. This latter issue can be overcome through the use of haploid cell lines [55]. Screening using genetrap insertional mutagenesis, within haploid human cells (HAP1), identified receptors for both Ebola and adeno-associated virus serotype 2 (AAV2), [39, 56]. In 2011, NCP1 was identified as a receptor for Ebola virus, accounting for 39 hits out of ~ 800,000 insertions. NCP1 was validated as a virus receptor by virus binding, replication and internalisation assays, and shRNA knockdown. In 2016, a library of mutagenized HAP1 cells containing almost all non-lethal knockouts was infected with AAV2 that expresses red fluorescent protein. Forty-six significant hits were identified from cells resistant to infection. Of these hits, a previously uncharacterized type I transmembrane protein, KIAA0319L, showed the greatest enrichment and was associated with 57 independent mutations. KIAA0319L was then confirmed as a receptor by generating CRISPR/Cas9 knockouts in eight different cell lines. All cell lines showed resistance to infection that was rescued through ectopic expression of KIAA0319L.

**Fig. 2:**
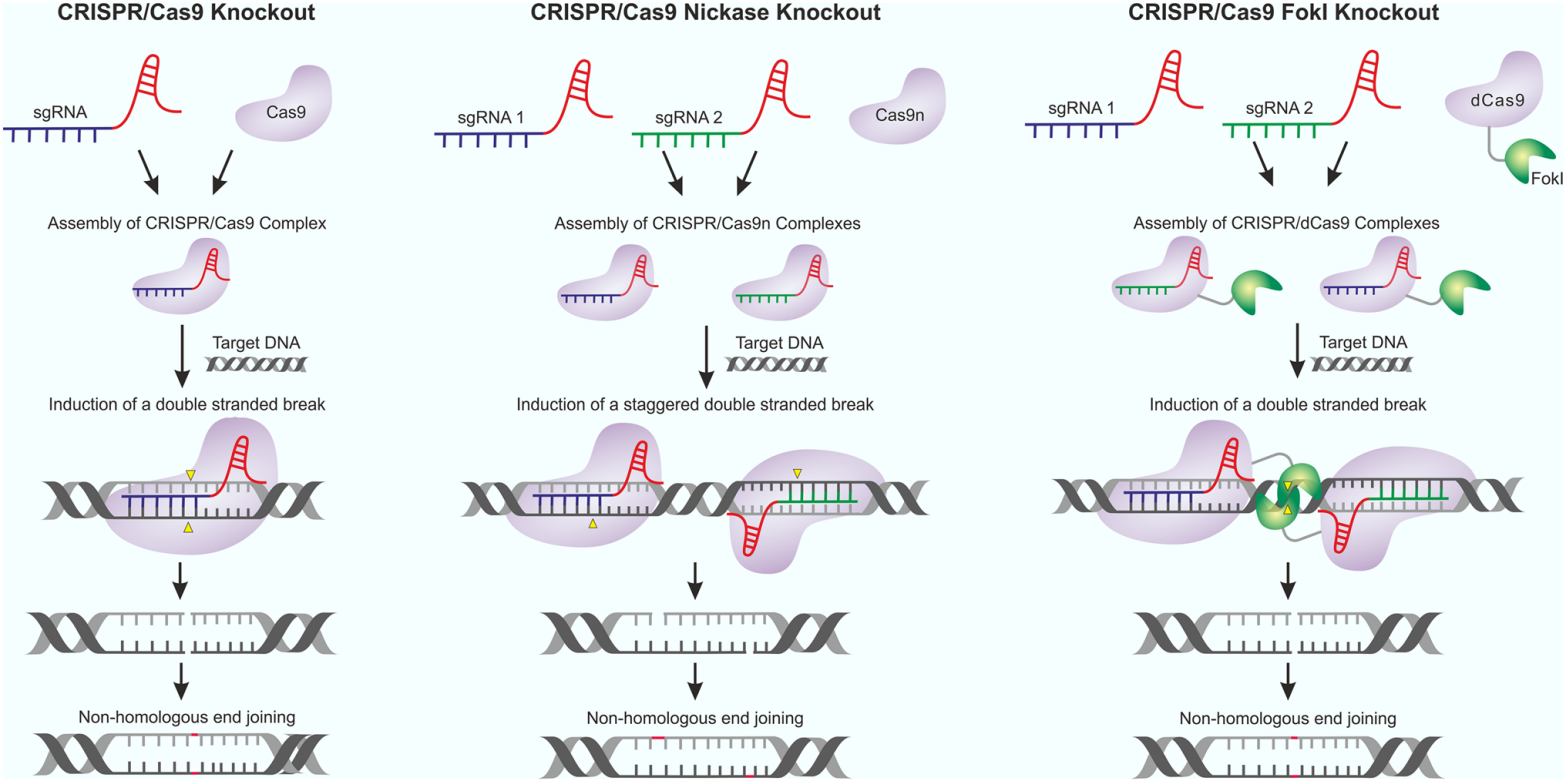
Schematic representation of CRISPR/Cas9, CRISPR/Cas9 nickase, and CRISPR/Cas9 *FOKI* genetic knockout mechanism. CRISPR/Cas9 genetic knockout: the CRISPR/Cas9 complex is first formed by combining an sgRNA and a Cas9 endonuclease. The sgRNA then guides the CRISPR/Cas9 complex to a specific region of the genomic DNA, and a double-stranded DNA break is induced by the Cas9 endonuclease. The double-stranded DNA break is then repaired non-homologously by the host cell, leading to the formation of insertions or deletions, which disrupt the open reading frame of the gene. CRISPR/Cas9 nickase genetic knockout: two CRISPR/Cas9 nickase complexes are formed with two separated sgRNAs and mutated Cas9 nickase endonucleases. The sgRNAs then guide the CRISPR/Cas9 complexes to specific regions of the genomic DNA, and two single-stranded DNA breaks are induced by the Cas9 endonucleases, forming a staggered double-stranded break. The double-stranded DNA break is then repaired non-homologously by the host cell. CRISPR/Cas9 *FOKI* genetic knockout: Two CRISPR/Cas9 *FOKI* complexes are formed with two separated sgRNAs and mutated dead Cas9 fused with the endonuclease *FOKI.* The sgRNAs then guide the CRISPR/Cas9 complexes to specific regions of the genomic DNA and the *FOKI* units dimerise. The *FOKI* dimer then induces a double-stranded DNA break which is repaired non-homologously

Site-specific mutagenesis can be performed using the CRISPR/Cas9 technology, which has the advantage that it equally targets all loci [57]. Although Cas9 is the most studied CRISPR associated protein, multiple CRISPR/Cas subtypes exist within prokaryotic organisms [58]. The CRISPR/Cas9 system evolved as an adaptive immune system within bacteria and archaea, employing a trans-acting CRISPR RNA: CRISPR RNA (tracrRNA:crRNA) duplex to target and destroy pathogenic DNA [59]. When performing genomic experiments, a fusion of the tracrRNA:crRNA duplex, known as a single-guide RNA (sgRNA) may be used. CRISPR/Cas9 knockouts are achieved through the generation of sgRNA targeted double-stranded DNA breaks, which, following error-prone non-homologous end joining, lead to the generation of null phenotypes (Fig. 2: CRISPR/Cas9 Knockout) [60, 61]. In 2019, receptors for both encephalomyocarditis virus and human cytomegalovirus (HMCV) were identified using genome-wide CRISPR/Cas9 screens with the GeCKOv2 sgRNA library. The library targets 19,050 genes with six sgRNAs per gene [62–64]. To identify receptors specifically required for HMCV infection in epithelial cells, Xiaofei et al. performed parallel CRISPR/Cas9 screens in ARPE-19 epithelial cells and HEL fibroblasts [63]. The multipass membrane protein, OR14I1, was identified as an initial hit exclusive to the epithelial cell screen, and validated as a viral receptor by shRNA knockdown followed by ectopic expression rescue, neutralisation, virus blocking and virus entry assays. Two identical CRISPR/Cas9 screens within HeLa cells identified nine initial hits associated with encephalomyocarditis virus infection in replicate pools. A disintegrin and metalloproteinase nine domain (ADAM9) was the most significant hit with all six sgRNAs being significantly enriched. ADAM9 knockouts were also shown to be resistant to virus infection in both primary lung fibroblasts (pLF) from mice and HEK293T cells. ADAM9 was confirmed to function as an entry receptor through the use of RNA bypass assays. In addition, ADAM9 was independently identified as a receptor for encephalomyocarditis virus, using genetrap mutagenesis screening in haploid cells [65]. This shows that genetic knockout screening is a robust technique for receptor identification (Table 1).

**Table 1:** Examples of identified cellular receptors with the identification method used

Virus	Identified receptor	Identification method	References
Sindbis virus	NRAMP	RNAi screening	[33]
Epstein–Barr virus	Ephrin receptor A2	RNAi screening	[34]
Ebolavirus	Niemann–Pick C1	Random insertional mutagenesis in Haploid cells	[39]
Adeno-associated virus (AAV) serotype 2	KIAA0319L	Random insertional mutagenesis in Haploid cells	[56]
Lassa virus	LAMP1	Random insertional mutagenesis in Haploid cells	[66]
Human-type A enteroviruses	kremen1	Random insertional mutagenesis in Haploid cells	[67]
NRP2	Lujo virus	Random insertional mutagenesis in Haploid cells	[68]
Encephalomyocarditis virus	ADAM9	CRISPR/Cas9 screening	[64]
Human cytomegalovirus	OR14I1	CRISPR/Cas9 screening	[63]
Norovirus	CD300If	CRISPR/Cas9 screening	[69, 70]
Arthritogenic alphaviruses	Mxra8	CRISPR/Cas9 screening	[71]
Andes virus and Sin Nombre virus	Protocadherin	CRISPR/Cas9 screening	[72]
Enterovirus B	Neonatal Fc receptor	CRISPR/Cas9 screening	[73]
Bat influenza virus	Human leukocyte antigen DR isotype	CRISPR/Cas9 screening	[74]
Hepatitis C virus	Claudin-1 and occludin	cDNA libraries	[8, 10]
Bombyx mori nucleopolyhedrovirus	SINAL10	cDNA libraries	[75]
Human cytomegalovirus	CD147	cDNA libraries	[76]
Japanese encephalitis virus	Hsp70	VOPBA	[77]
Old World arenaviruses	α-Dystroglycan	VOPBA	[78]
Human cytomegalovirus	Nrp2 and PDGFRa	Protein microarrays	[79]
New world arenaviruses	Transferrin receptor	Affinity capture mass spectrometry	[80]
Nipah virus	Ephrin B2	Affinity capture mass spectrometry	[81]
Japanese encephalitis virus	PLVAP and GKN3	Affinity purification–mass spectrometry	[82]
Vaccinina virus	AXL, M6PR, DAG1, CSPG4 and CDH13	Cross-linked mass spectrometry	[83]

In contrast to RNAi, CRISPR/Cas9 knockouts have been shown to produce fewer false negative results [84]. Additionally, CRISPR/Cas9 knockout screens have been developed within haploid cell lines, therefore increasing the reliability of generating gene disrupted phenotypes [85]. Due to tolerated mismatches at the sgRNA–target site interface, false positives also occur using CRISPR/Cas9 but at a 34% lower rate than with RNAi [86–88]. False positives can be reduced through the use of truncated sgRNAs (17–18 nucleotides compared to 20 nucleotides), which have a lower DNA-binding affinity and, therefore, are unable to tolerate mismatches in the target DNA [89]. Additionally, mutant CRISPR/Cas9 systems which require the correct localisation of two CRISPR/Cas9 monomers to induce gene knockouts can also limit off-target effects. This approach is exemplified by CRISPR/Cas9 nickases, where the Cas9 molecule has been mutated to only introduce single-stranded DNA breaks, and therefore, two closely spaced CRISPR/Cas9 complexes are required to introduce staggered double-stranded DNA breaks (Fig. 2: CRISPR/Cas9 Nickase Knockout) [90]. As single-stranded nicks are repaired with high fidelity by base excision repair, this minimises the generation of off-target gene depletions [91]. In another approach, the Cas9 nuclease activity has been removed and the endonuclease domain of *FokI* fused to the Cas9. This mutant CRISPR/Cas9 system can only induce double-stranded DNA breaks when two *FokI* endonuclease domains undergo dimerization, as *FokI* is not active in its monomeric form (Fig. 2: CRISPR/Cas9 FokI Knockout) [92]. Although the utilisation of nickases or Cas9 *FOKI* can reduce off-target effects, they also possess challenges for delivery with viral vectors, due to their increased nucleic-acid payload size. Therefore, the development of higher fidelity variants of Cas9 through protein engineering is favourable. Several such high fidelity Cas9s have now been developed including: SpCas9-HF1 [93], Sniper-Cas9 [94], and eSpCas9 [95].

## Ectopic expression of complementary DNA

In contrast to RNAi, random insertional mutagenesis, and CRISPR/Cas9 knockouts, cDNA libraries are used to introduce new functionalities into cell lines. cDNA libraries are synthesised from mRNA using reverse transcriptases, before being cloned into plasmid or lentiviral expression systems [96–98]. These libraries can then be utilised in GOF screening, whereby cDNA libraries developed from susceptible cell lines are transduced into non-susceptible cell lines, and retested for susceptibility. cDNA libraries have been used for receptor identification since the 1990s [9, 75, 76]. In 2018, a cDNA library containing 8.4 × 10^6^ primary clones derived from ARPE-19 epithelial cells was used to identify genes that promote HMCV entry [76]. HeLa cells which have a low HCMV infection rate of 1–5% were transfected with the cDNA library and infected with HMCV that expresses green fluorescent protein (GFP). Approximately 100 out of 1 million clones showed an increase in green fluorescence, indicative of increased infectivity. DNA sequencing of the GFP expressing clones identified the cell surface molecule CD147 as a potential receptor. This was confirmed by shRNA knockdown, HMCV entry assays and co-localisation immunofluorescence. GOF and LOF approaches are strongly complimentary, with cDNA rescue experiments acting as a major verification method for hits identified in RNAi screening (reviewed in [99]).

There are, however, several drawbacks to consider when using cDNA libraries for GOF screening. Firstly, it may be difficult to obtain a suitable non-susceptible cell line. Second, non-susceptible cell lines must be genetically manipulable. Third, the number of copies of each gene within the cDNA library is potentially biased by the abundance of mRNA in the susceptible cell line at the time of isolation. Therefore, receptors which have low expression levels may not be represented in cDNA libraries, preventing their identification. Fourth, cDNA libraries may contain truncated cDNA clones arising from early termination of reverse transcriptase activity. The inclusion of truncated cDNAs in the library can be prevented using high efficiency cloning methods which isolate full-length cDNAs using two dimensional electrophoretic separation [100]. Finally, the reverse transcriptase step is error prone, and therefore, unwanted mutations within the cDNA library may lead to false negatives [101].

## Interactomics and proteomics

In contrast to the previously described genomic approaches, interactome and proteomics aim to directly identify the protein–protein interactions (PPIs), which occur between virus attachment proteins and their host receptors. Historically, this has been performed using virus overlay protein binding assays (VOPBA) (reviewed in [102]). Although VOPBA have successfully identified receptors for several viruses, including lymphocytic choriomeningitis virus and lassa virus [78], VOPBA suffer from several problems. In VOPBA, all cellular proteins are subjected to gel electrophoresis, transferred onto a membrane, and then probed with virus. Bands where virus–receptor interactions occur can then be visualised with antibodies, and the corresponding protein in a duplicate gel identified using mass spectrometry. Failure to identify receptors can occur if the necessary three-dimensional conformation of the attachment domains or receptor complexes is destroyed by SDS-PAGE. Furthermore, it may not be possible to detect virus–receptor interactions which have low binding affinities [103]. More recent methods remove the need for gel electrophoresis, instead probing virus–receptor interactions in more native environments, overcoming some of the issues present by VOPBA. Here, we will discuss the use of protein microarrays, affinity purification–mass spectrometry (AP–MS), and cross-linked mass spectrometry (XL-MS) for receptor identification.

## Protein microarrays

Unlike VOPBA protein microarrays allow for the direct detection of PPIs without the requirement of SDS-PAGE. First, a protein microarray is generated by immobilising purified ‘bait’ proteins onto a glass slide. Next, the microarray is probed with virus, and the PPIs detected through the use of fluorescently-, enzymatically-, or radio-labelled recombinant proteins within the array, surface plasmon resonance imaging, atomic force microscopy, electrochemical impedance spectroscopy, and mass spectrometry (reviewed in [104]).

The first functional protein array was developed by Zhu et al. in 2001 and used to determine substrate specificity within the yeast proteome [105]. Since then, many protein microarrays have been developed, including several commercial microarrays (reviewed in [106]). Nevertheless, protein microarrays which target extracellular receptors remain few and far between. This can be partially attributed to the difficulties associated with the production of soluble transmembrane proteins. In 2007, Bushell et al. developed an avidity-based extracellular interaction screen (Avexis), where they recombinantly expressed the extracellular domains of transmembrane proteins, removing the insoluble transmembrane region [107]. To identify low-affinity interactions, the proteins were additionally tagged with a coiled-coil sequence from the rat cartilage oligomeric matrix protein. This causes the pentamerization of the ‘bait’ proteins and increases binding avidity.

In 2018, a high-throughput screening approach, based on Avexis was utilised for the identification of HMCV–receptor interactions [79]. A library containing the ectodomains of 1297 human single transmembrane receptors was set up on an automated, cell independent platform. The library was then probed with the HMCV envelope glycoprotein pentamer, gHgLpUL128-131A, and trimer, gHgLgO. Three hits were identified for the trimer: platelet-derived growth factor receptor A (PDGFRa), transforming growth factor beta receptor type 3 (TGFbRIII), and neuregulin2 (NRG2), and four hits for the pentamer: neuropilin-2 (Nrp2), thrombomodulin (THBD), the leukocyte immunoglobulin-like receptor (LILRB3), and the immunoglobulin alpha Fc receptor (FCAR). The interactions were then probed using biolayer interferometry and surface plasmon resonance. No interaction could be detected for NRG2 or FCAR, and only weak binding to LILRB3; therefore, these hits were excluded from further studies. The remaining potential receptors were then validated using competition assays with both recombinant protein and anti-receptor antibodies, lentiviral overexpression, and CRISPR/Cas9 knockdown. It was determined that only Nrp2 and PDGFRa acted as functional receptors in vivo. Although THBD and TGFbRIII interact with HMCV in vitro, they were not shown to be essential for virus entry.

Although protein microarrays have the ability to detect PPIs in vitro, the detected interactions may not be biologically relevant in vivo. This is mainly due to difficulties associated with producing recombinant proteins, which possess native post-translational modifications, such as glycans or disulphide bonds. Another limitation of protein microarrays is the cost and time requirements associated with generating large-scale libraries of recombinant proteins. A method to overcome this is the use of nucleic-acid programmable protein arrays (NAPPA), where DNA is directly deposited onto the microarray and the proteins are synthesised in situ using in vitro transcription and translation (IVTT) [108]. In 2016, a proof-of-concept study utilised IVTT and microfluidics to create an array of ~ 2100 proteins from ~ 2700 linear synthetic genes [109]. The array was then probed with simian virus 40 (SV40) and 99 interactions were observed, including 14 of the 22 positive controls. This demonstrates a high false-negative discovery rate of 36%. Finally, 25 hits were subjected to validation by co-immunoprecipitation, which confirmed 18 hits.

## Affinity purification–mass spectrometry

**Fig. 3:**
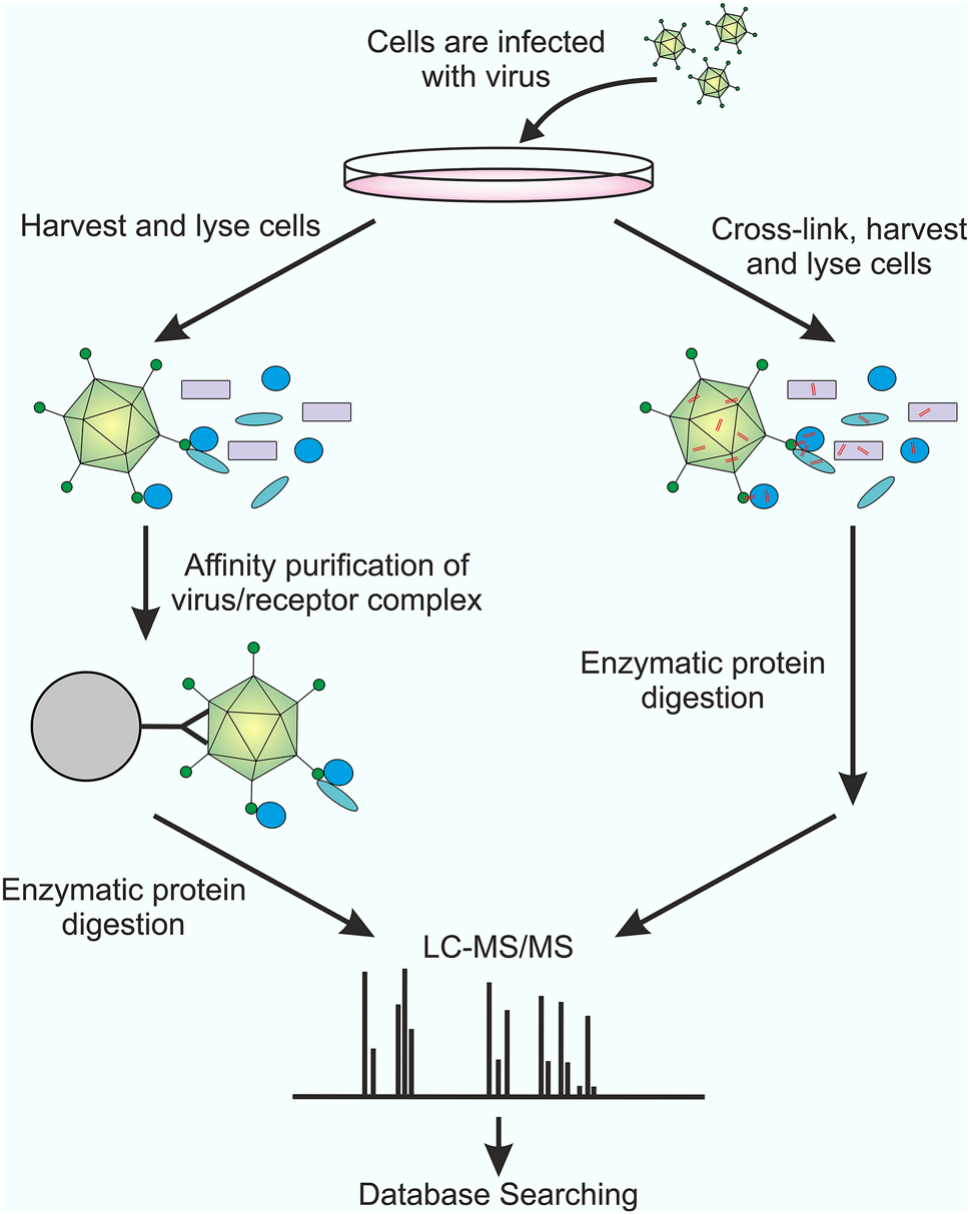
AP–MS and XL-MS workflow comparison. AP–MS workflow: cells are first infected with the virus of interest, harvested, and lysed. The virus–receptor complexes are then isolated using affinity purification, and the purified proteins enzymatically digested and identified using LC–MS/MS and database searching. In XL-MS, the cells are also first infected with virus. However, a chemical cross-linking reaction is then performed, before the harvesting and lysis of the host cells. Next, the proteins are enzymatically digested and identified using LC–MS/MS and database searching

AP–MS is one of the most common proteomic approaches used for the identification of virus–host interactions. The AP–MS, workflow is as follows: first, the host cells are infected with virus, allowing for the formation of virus–receptor complexes. Following cell lysis, the complexes are then isolated by affinity purification. Finally, the complexes can be identified and quantified using mass spectrometry (Fig. 3).

Often affinity capture with antibodies has been used to isolate virus–receptor complexes prior to analysis by mass spectrometry [110]. This requires a high affinity, specific antibody, preferentially recognising a conformational epitope of the virus capsid that is not hidden by receptor binding. Alternatively, affinity-tagged viruses can be used [111, 112]. This method enables binding partners against the affinity tag to be used in the affinity purification step. Therefore, tags can be chosen for which high affinity binding partners are readily available. To produce affinity-tagged virus, the virus either needs to be genetically tractable (e.g., through reverse genetics) to introduce the tag, or the affinity-tagged protein is incorporated through in *trans* expression of the gene during virus infection. Again, the tag insertion should not sterically hinder receptor binding. Although not used to identify viral receptors, this approach has been used to identify virus–host interactions, involved in replication within dengue virus serotype 2 (DENV-2) [113]. Here, the full-length DENV-2 RNA genome containing a poly-histidine and FLAG-tagged NS5 was synthesised in vitro using T7 RNA polymerase. Huh-7 cells were then infected with tagged-DENV-2, and the NS5 complexes isolated via FLAG pull-down 48 h post-infection, leading to the identification of 97 interacting proteins. The most common AP–MS approach used in the identification of viral receptors is the use of individually tagged recombinant viral glycoproteins. The approach was used recently in 2018 to identify host cell receptors for Japanese encephalitis virus (JEV) [82]. Affinity purification was performed using plasma membrane fractions of BALB/c mouse brain and the recombinant expressed, his-tagged, envelope protein of JEV, identifying 42 interacting proteins. Hits were then analysed for mRNA upregulation during JEV infection and the envelope–host protein interactions studied in silico. Two hits, plasmalemma vesicle-associated protein and gastrokine three were then validated as viral receptors by overexpression using cDNA and knockdown using siRNAi. Although frequently used, this approach may not faithfully reproduce receptor-binding sites due to the exposure of surfaces that are hidden within virus assembly, therefore leading to the identification of false positives.

False positives can also occur during AP–MS experiments due to the co-isolation of non-specifically bound proteins. The presence of non-specifically bound proteins can partially be reduced through the use of tandem affinity purifications [114]; where two different epitope tags are incorporated into the recombinant virus, and the virus–receptor complexes are purified in two consecutive affinity purification steps.

## Cross-linking mass spectrometry

The interactions between viral attachment proteins and host receptors are intermolecular and transient; therefore, their direct detection within native environments is challenging. Cross-linking can overcome this, by introducing covalent linkages between the viral attachment protein and the host receptor. In the early 1970s, cross-linking was used in conjunction with gel electrophoresis, to identify PPIs within ribosomes [115]. More recently, cross-linking has been combined with high-resolution mass spectrometry (XL-MS), to identify PPIs and their physical interaction contacts [83].

During XL-MS, interacting proteins are covalently tethered using chemical cross-linkers. Following cross-linking, the proteins are enzymatically digested, resulting in a complex peptide mixture. This peptide mixture is then analysed using liquid chromatography–tandem mass spectrometry (LC–MS/MS) and the cross-linked peptides identified by comparing experimental MS/MS spectra, against a database of computationally generated theoretical spectra. As the length of the cross-linkers is known, these can be used as distance constraints in order to perform structural modelling [reviewed in [116]].

There are numerous different types of cross-linkers available including: homobifunctional cross-linkers, heterobifunctional cross-linkers, zero-length cross-linkers, and trifunctional cross-linkers [reviewed in [117] ]. Trifunctional cross-linkers are particularly useful, as they allow for the introduction of additional functionalities, such as biotin handles, which can be used for affinity purification of the cross-linked species following protein digestion [83, 118]. Furthermore, trifunctional cross-linkers can also incorporate chemical cleavage sites to remove the biotin moiety following purification and an isotopically labelled spacer arm, which can be used for quantitation [119]. Frei et al. [120] performed a proof-of-concept study, whereby they identified seven cell surface proteins as potential virus receptors for mature vaccinia viruses, using a novel trifunctional chemoproteomic reagent (TRICEPS) in XL-MS. The TRICEPS reagent contains three functionalities: an NHS ester for coupling to ligands via primary amines; a trifluoroacetyl-protected hydrazine that can bind glycoproteins on the cell surface following the introduction of aldehydes via mild oxidation, and a biotin group for affinity purification of the cross-linked products. To validate the hits, the proteins were subjected to siRNA knockdown and five proteins AXL, M6PR, DAG1, CSPG4, and CDH13 were shown to reduce virus infectivity by 40–60%. Furthermore, three of the proteins identified: CSPG4, DAG1, and AXL had previously been identified as virus receptors or attachment factors using competition assays, mutagenesis studies, and co-localisation experiments [121–123]. Despite the success of this proof-of-concept study, no further virus receptors have been identified using XL-MS. A possible cause for this is the complexity of data analysis associated with XL-MS (reviewed in [124]). MS/MS fragmentation is complicated for cross-linked peptides, as the resulting peptide contains fragments from two proteins. This leads to a higher precursor charge state and a greater number of fragment ions. Furthermore, four types of cross-linked peptides can be present in the sample: dead-end cross-links, intrapeptide cross-links, interpeptide cross-links, and higher order cross-linked peptides. Consequently, to identify peptides, all possible peptide–peptide combinations need to be considered when performing database searches, drastically increasing the search space. Several specialised algorithms and software packages have been developed, which can aid in the identification of peptides (reviewed in [124]). We expect that such algorithm adaption will lead to more discoveries in the near future.

## Practical considerations

### Genetic screening approach

There are two types of screening approaches which can be adopted: pooled and arrayed, with each method possessing a unique set of advantages (Table 2). In pooled screening, a population of cells is either transduced with an RNA or cDNA library (RNAi, CRISPR/Cas9, and cDNA), or subjected to retroviral gene trapping (haploid cell knockouts). The pooled cells then undergo selection for transduction or retroviral incorporation, and the populations are expanded. Following expansion, the cells are infected with the virus in question, and enrichment is performed either looking for a GOF or LOF that has an effect. Finally, hits are identified by next-generation sequencing [125–127].

**Table 2:** Advantages and disadvantages of pooled and arrayed genomic screening approaches

	Pros	Cons
Arrayed screening	Can select a gradation of phenotypes Rapid identification using library key Short-term screening (less than 10 days) Can be used for high-throughput screening Custom-made libraries are available	For haploid cells, this requires long-term culturing and storage of numerous cell lines Attached cells only Expensive to purchase, use, and maintain libraries Requires high-throughput plate reader or microscope for analysis
Pooled screening	Simple setup Does not require any specialised equipment Can be used with suspension cells Long-term screening (greater than 10 days) Lower cost, particularly when performing survival screens	PCR/next-generation sequencing needed to identify hits Cannot perform high-throughput screening Requires high cell number Requires a selection step Limited number of readouts

In arrayed RNAi, CRISPR/Cas9, and cDNA screening, cells are also transduced with an RNA or cDNA library [128]. However, in contrast to pooled screening, a single gene is targeted in each well, of a multi-well plate. In arrayed, random insertional mutagenesis screening, knockouts are first generated within low cell density pools; the individual colonies are then picked and cultured in multi-well plates [129]. Following knockdown or ectopic expression, the cells are infected with the virus in question, and the viral replication or cell number monitored for a GOF or LOF effect. Finally, hits are identified using the library key.

### Choice of cell line, tissue, or animal model

Viruses demonstrate varying host ranges and tissue tropisms. Whether or not a cell is susceptible to infection is dependent on the expression of suitable entry receptors on the cell’s surface. However, some viruses may utilise different receptors depending on the cell type, allowing them to be more promiscuous. Therefore, receptors identified within a specific haploid or diploid cell line, are not necessarily representative of all the receptors used by the virus, within all hosts, or all host tissues. This has been demonstrated for adenovirus serotype 5, where the coxsackievirus and adenovirus receptor (CAR) has been shown to act as the primary receptor in vitro. However, the vitamin K-dependent coagulation factor X (FX) acts as an alternative receptor within the human liver, bypassing CAR, and initiating infection [130, 131]. Furthermore, some viruses have been shown to adapt to cell culture conditions allowing them to utilise new receptors such as heparin sulphate [132]. The use of 3D cell models and animal models may lead to the identification of different receptors to those found in standard cell culture [133].

## Conclusions

The study of virus–receptor interactions is crucial for the development of antiviral drugs, vaccines, and new diagnostic technologies. Nevertheless, receptor identification has often been a laborious and time-consuming process. High-throughput approaches now provide alternative, potentially faster methods for host receptor discovery, and have been used to identify numerous receptors within recent years (Table 1). In particular, genetic knockout screening has grown in popularity, leading to the discovery of several bona fide new receptors since 2017, including: NRP2 for Lujo Virus, protocadherin for Andes virus and Sin Nombre virus, kremen1 for multiple human-type A enteroviruses, neonatal Fc receptor for enterovirus B, and human leukocyte antigen DR isotype for bat influenza viruses [67, 68, 72–74].

Despite numerous elegant studies, our knowledge of entry receptors still remains limited. This can be linked to a series of experimental challenges including: the use of relevant cell lines, tissues or animal models; the production of suitable virus strains, genetic tractability, and the identification of interactions in native environments (Tables 2, 3). Many of these methods result in multiple candidate molecules being identified and these need to be narrowed down in further validation studies. The choice of validation method depends on the initial identification method. Often orthogonal experiments are used, e.g., proteomic approaches are used to validate results from genomic approaches and vice versa. However, several other approaches can be used to validate virus receptors, such as, competition assays [134], small molecule inhibition of binding [135], co-localization immunofluorescence microscopy [39], and Förster resonance energy transfer assays [136].

**Table 3:** Advantages and disadvantages of proteomic approaches

	Pros	Cons
Protein microarrays	Several microarrays available commercially Low avidity binding detected using ‘bait’ protein pentamerization Quick generation of large-scale libraries using nucleic-acid programmable protein arrays Several methods used to detect interactions	Detected interaction may not be biologically relevant in vivo Difficult to produce recombinant proteins, with native post-translational modifications Time-consuming and expensive to generate libraries of recombinant proteins High false-negative discovery rate in nucleic-acid programmable protein arrays
Affinity purification–mass spectrometry	Affinity tagging allows the study of proteins where native antibodies are not available Use of affinity tags that have high affinity antibodies readily available Library-independent method allows for true genome-wide high-throughput capability Proteins purified in native form	Introduction of affinity tag requires a genetically tractable virus. Affinity tag may interfere with virus function and protein folding Cell lysis and affinity purification may prevent the detection of low avidity and transient interactions. False positives due to co-isolation of non-specifically bound proteins
Cross-linked mass spectrometry	Identification of low avidity and transient interactions Enrichment and quantitation using trifunctional cross-linkers Library-independent method allows for true genome-wide high-throughput capability Can gain additional structural information about binding site	Requires specialised software to identify cross-linked peptides Requires the presence of residues susceptible to cross-linking on the surface of the virus and receptor Detected proteins may not be functional receptors in vivo

In summary, receptor identification is seldom a straightforward process. Therefore, a one-method-fits-all approach cannot be adopted for receptor identification. More commonly, receptors are identified using a combination of different approaches, which contribute to the overall picture of virus entry.
